# Promoting Integrated Care through a Global Treatment Budget: A Qualitative Study in German Mental Health Care using Rogers’ Diffusion of Innovation Theory

**DOI:** 10.5334/ijic.5940

**Published:** 2021-11-30

**Authors:** Farideh Carolin Afraz, Amyn Vogel, Carsten Dreher, Anne Berghöfer

**Affiliations:** 1 Institute of Social Medicine, Epidemiology and Health Economics, Charité – Universitätsmedizin Berlin, Luisenstr. 57, 10117 Berlin, Germany; 2 Department of Information Systems, School of Business and Economics – Freie Universität Berlin, Garystr. 21, 14195 Berlin, Germany; 3 Professorship of Innovation Management, School of Business and Economics – Freie Universität Berlin, Thielallee 73, 14195 Berlin, Germany

**Keywords:** global treatment budget, flexible, integrated treatment models FIT, cross-sectoral care, Rogers’ innovation diffusion theory, qualitative study, qualitative content analysis

## Abstract

**Introduction:**

Since 2003, as a means of enabling integrated care the German mental health care system has offered the innovative option of agreeing a Global Treatment Budget (GTB, also known as a regional psychiatric budget or innovative flexible and integrative forms of treatment FIT) with health insurers and regional care providers across sectors. Despite promoting legal frameworks and positive evidence on improving quality of patient care, this model has not spread widely. The aim of this study is to identify inhibiting and facilitating factors for the innovation diffusion.

**Theory and methods:**

We conducted expert interviews with 19 actors from nine German regions involved in GTBs, using a self-developed questionnaire based on Rogers’ theory on innovation diffusion extended by the innovation system approach. Interviews were analysed applying qualitative content analysis. Code categories were built deductively operationalising Rogers’ theory and inductively from the data generated.

**Results:**

Observability of the innovation was perceived as good, but trialability, reversibility, compatibility with regular care structures as low, and thus the perceived risks of adoption as high. Complexity up to implementation is high, caused by numerous individuals and stakeholder groups involved. Diffusion took place in environments of strong individuals with venturesomeness, opinion leadership, and informal networking. As favourable framework conditions the monopoly and non-profit position of hospitals in well-defined care regions were identified.

**Discussion and Conclusions:**

Diffusion of integrated care could be accelerated by dissolving the multi-actor constellation, changing the communication strategy, and adapting the legal framework.

## Introduction

The development of integrated care and patient-centred models in the German mental health care system lags far behind political and stakeholders’ vision [1,2]. Complexity of provider structure is considerably high; hence patients are often overstrained by clinical pathways resulting in reduced adherence and outcome of therapeutic interventions [3,4]. Accordingly, the fragmentation of sectors and their separate financing have been recognised and addressed recently as a major obstacle to a more patient-centred mental health care in Germany [5,6].

Various efforts have been made to establish integrated care by pursuing selected integrated care contracts and nationwide disease management programs [7]. However, the aim to improve cooperation in mental health care was not met to a large extent [8,9,10].

**Figure 1:**
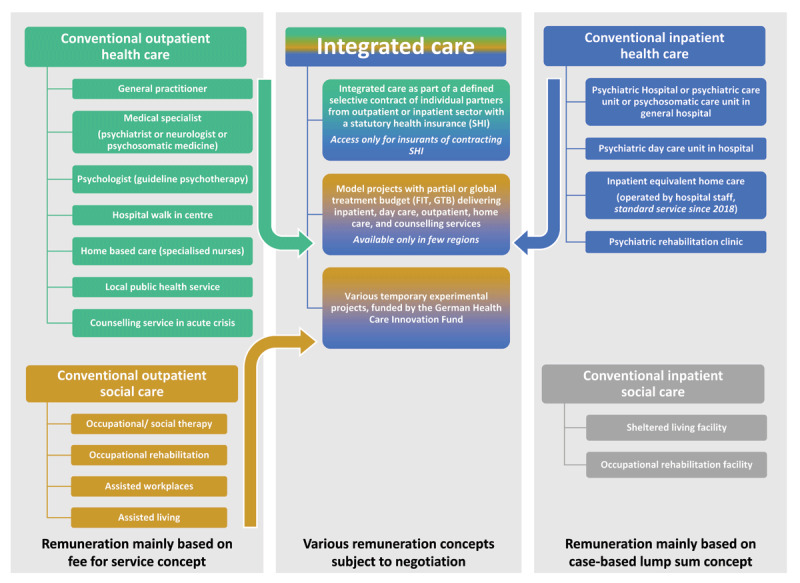
Agenda of mental health and social care in Germany. Various providers in the outpatient sector mainly paid on a fee for service concept, providers in the inpatient sector mainly paid by case-based lump sums. Innovative models of integrated care include services from outpatient and inpatient health care and partly from outpatient social care. Models with global budgets framed black (own figure adapted from [4,13]).

The present study focuses on one innovative care and funding model, the Global Treatment Budget (GTB), intentionally created by the legislator to strengthen particularly cross-sectoral care of mental illnesses and improve patient care. The legislator pushed innovative models intending to replace long inpatient stays, as current treatment is often characterised by comparably long duration due to chronicity, repeated contacts and a particularly high number of different providers involved (***Figure 1***). To this end, GTB allows – as capitation principle based on the expected number of annually treated patients – an alternative way of funding in a defined region [11,12]. It allows for establishing sustainable flexible and continuous cross-sectoral care tailored to individual patient needs from a single hand, in a sense, through one joint budget across all psychiatric treatment settings. Thus, jointly agreed contracts of GTB, supporting community-based psychiatric care approaches, fulfils the expectations of the explanatory memorandum of the law by softening the fronts between hospital and statutory health insurance (SHI) and between inpatient, day-clinic, outpatient, and home treatment care. At the same time, it allows efficient treatment as it is independent of misaligned remuneration incentives.

In Germany first GTBs (formerly known as regional psychiatry budgets) as a means of funding to enable integrated mental health care were established in 2003 based on contracts [14]. Since 2012 possibilities for integrated care have been expanded to facilitate integrated care approaches [15] as a wider legal frame enabled the implementation of further GTBs in psychiatry [16,17,18,19]. Evidence of hitherto implemented GTBs enabling integrated care shows various advantages when compared to regular mental health care, e.g. in patient compliance, improved patient-relevant outcomes [12,17,19,20,21,22,23] as well as improved working conditions for medical staff [16,19,24,25,26,27] and cost effectiveness [28]. Despite the above-mentioned overall positive experiences to date and the evidence for manifold improvement of mental health care, the GTB has not prevailed in mainstream mental health care in Germany; after more than 15 years of testing, less than 5% of care regions have followed this model [13]. The sluggish implementation of complex new health care models is not new; it is attributed to the models’ complexity per se, path dependencies in the health care system, and lack of governance knowledge [29,30].

Beyond these system-level explanations, specific reasons why this particular model does not diffuse have not been explored yet. Therefore, an investigation using Rogers’ model of innovation diffusion and a qualitative research approach seemed reasonable to explore promoting factors and major obstacles occurring in diffusion processes of this innovative model of integrated mental health care.

## Theory and methods

### Theoretical framework

To analyse innovation diffusion in health care, established models such as Rogers’ diffusion theory [31,32] will be coupled and intertwined with the analysis of innovation system dynamics [33,34]. These different perspectives combined allow for capturing the complexity of the diffusion processes and the multi-level challenges.

Rogers’ central question is how innovations spread in social systems and which factors influence diffusion. His paradigm of the adoption process covers characteristics of actors (e.g. values, skills, status), situational perception (e.g. social norms, economic constraints), perceived characteristics of the innovation itself (advantage, compatibility, complexity, divisibility, and communicability), and information sources [31]. Thus, factors at individual and system level as well as the communication process are included [32,35]. In Rogers’ more recent work [32], he issues for adopters the problem of collective decisions for or against an innovation. In contrast to individual decisions, such as in the consumer goods sector, or authoritarian decisions, such as legal orders, actors with diverging interests are involved here. Rogers’ theory has been successfully applied to analyse diffusion processes in health care [36]. Regarding the influence on adoption and adoption speed, there is indeed still an open research question even with Rogers.

Rogers’ theory is one of the most prominent models on the diffusion of innovations in social sciences. Compared to other diffusion models based on other science traditions, it includes a full set of criteria for the individual adoption decision (whether by persons or organisations). Models out of the epidemiological tradition and medical sciences are using big data sets and exploring the underlying mathematical functions in order to make predictions for further developments (e. g. predicting the spread of infectious diseases). Economic models, like the Mansfield diffusion model [37] or the probit-family of diffusion models [38], are looking at the individual adopter decision, but using only aspects like profitability and complexity of predominantly technical process innovations. Very popular recent models of the multilevel perspective [39] characterise societal transition processes by investigating the interdependencies of macro-, meso-, and micro-levels, but they refrain from explaining individual adopter’s decision. In our case, we have the combination of looking at a social practice implementation instead of a technological innovation and in a mainly non-profit environment of a single organisation. Instead of hard technologies involved, a complex interplay between external factors, as legislation, hard and soft institutional settings, social behaviour, and individual attitudes of various actors involved in the individual decision of a hospital takes place. Rogers’ model at best encompasses these aspects and provides the guiding questions and criteria for investigation without imposing too many restrictions as by the other models of diffusion.

To analytically penetrate the involved networks or collectives, the innovation system approach is additionally useful for the diffusion of innovations [34,40]. This approach analyses actors active in a specific innovation field, their interactions in knowledge building and learning processes, additionally the identification of their relevance for the adoption. This closes the gap of ruptures approach for collective decision-making.

### Methods

The study was designed as a cross-sectional study based on qualitative interviews with stakeholders involved in past GTB-negotiations. Results were triangulated with documents and publications.

#### Interview guide

To carry out semi-structured interviews an interview guide was developed based on Rogers’ innovation diffusion model [32], systematically inquiring about the aspects considered important by Rogers (FCA and AV). The guide was finalised in an iterative team process (FCA, AV, CD, and AB), and adapted to the experts’ roles (professional group, status of adoption). The interview guide focused on the main categories from Rogers extended by categories from the innovation system approach (supplementary table 1). Results of a pilot interview led to editorial refinement, thus was included in the analysis. Interviews were carried out between July 2018 and April 2019 (FCA, AV, and AB) and lasted one hour on average. Recorded interviews were transcribed and pseudonymised, anchor examples translated to English.

#### Sampling and field access

The sample of experts comprised directors of psychiatric hospitals (perspective of psychiatrists, P), representatives of commercial management (C) of the same hospitals and representatives of SHIs (I) responsible for GTB-negotiations in the corresponding region. Thus, all actors in the innovation process affecting the collective decision-making were represented.

Respondents were identified through dialogue with members of the German network of model project participants (AB) and their personal recommendations via snow balling principle. Sample size of 24 interviews was chosen initially. Four groups of local experts comprised: Early adopters, late adopters, failed adopters, and observers from two regions each. Groups emerged from structural data and literature. The number could be adapted depending on the theoretical saturation by the interviews.

#### Data analysis

Data analysis followed the methods of Mayring’s summarising qualitative content analysis [41]. Against the background of Rogers’ established theory, we thought this was the appropriate method. Primarily the interview transcripts were analysed with a set of deductively assigned code categories, based on criteria guided by Rogers’ theoretical framework. The categories were enriched with code categories inductively determined from the material and inducing new aspects beyond Rogers’ theory (supplementary table 2). Computer aided data analysis was performed with MAXQDA, reduction and abstraction steps in Microsoft Excel. Coding reliability was assessed and improved several times through independent coding by two different coders (FCA, AB). Results were discussed within the group regularly to ensure intersubjectivity (FCA, AV, CD, and AB).

## Results

In total 19 local experts were successfully recruited, 14 declined participation for various reasons (lack of authorisation, subjective lack of competence in the subject, lack of time) (***Table 1***).

**Table 1:** Structure of the sample interviewed.


	PSYCHIATRISTS (P)	REPRESENTATIVES OF COMMERCIAL MANAGEMENT (C)	REPRESENTATIVES OF SHIS (I)	WILLINGNESS FOR INTERVIEW (INTERVIEWS CARRIED OUT)

Early Adopters	2	5	4	55% (6)

Late Adopters	3	1	5	56% (5)

Failed Adopters	4	3	2	89% (8)

Observers	4	0	0	0% (0)

Performed interviews	62% (8)	67% (6)	45% (5)	58% (19)


Initially, four case categories should be included, represented by two health care regions each (supplementary table 3). Presumed observers turned out in interviews to be failed adopters at various stages of negotiations. The number of interviews of adopters and failed adopters was adapted because of theoretical saturation [42]. Extending the field to recruit observers in the regional neighbourhood was not successful. Additional informal conversations could be held, which were explicitly not released for analysis.

A total of 2,097 relevant statements were identified and assigned to 164 codes.

### Stages of Innovation Decision Process

Rogers’ five stages of innovation decision process – consisting of knowledge, persuasion, decision, implementation, and confirmation – could be identified in the GTB-diffusion. Especially psychiatrists have sufficient awareness of the GTB as funding option for integrated care, knowledge of how it works in principle, and how to implement it in practice. They remarked to have achieved their knowledge by intensive personal exchange, mutual visits, and various medical-scientific publications and lectures–driven significantly by committed and respected personalities representing opinion leaders according to Rogers.

Psychiatrists stated that they initiated most negotiations and were subsequently supported by their controllers. In most cases, different stakeholder groups agreed, there had been a lack of SHIs appreciating GTB-implemention, severely limiting the total number of negotiations taken place.

Psychiatrists noticed that the later GTB-contracts were agreed upon, the more complicated it became to find consensus view among various SHIs. Irrespective of outcome, most respondents, especially hospitals, experienced negotiations as distinctly time-consuming and labor-intensive. They expressed that despite initial consensus on desire for an integrated care model, insurmountable interest conflicts regarding contract details sometimes led to failure. Moreover, hospitals would have no claim by law to contract an alternative funding option like a GTB.

Participants explained that confirmation of the pro-GTB-decision took place both at the level of medical-scientific publications, driven by evaluation provided for in the current legal framework, and personal exchange, mainly within psychiatric network activities. Finally, none of the adopter regions cancelled or did not renew their GTB-contracts after expiry, suggesting at least a basic level of satisfaction.

### Perceived Characteristics of the Innovation

#### Relative Advantage

Psychiatrists perceived many advantages of integrated care associated with GTB, focussing primarily on medical-therapeutic aspects such as greater flexibility in treatment spectrum with inter alia new and not yet offered services, less bureaucracy and greater sustainability of patient-specific therapies, closely oriented to the patient‘s life and characterised by continuity of treatment. Misaligned financial incentives of traditional inpatient remuneration based on highest possible bed occupancy would be replaced by positive incentives regarding long-lasting therapeutic success.

Mainly psychiatrists reported that in addition to increased patient satisfaction, job satisfaction of various hospital staff was higher in integrated care models enabled by GTB.

Controllers agreed that patient outcomes improved. They noted that their director psychiatrists used to convince them of the care advantages before entering GTB-negotiations. However, the economic assessment varied widely among the controllers. Reported spectrum ranges from potential cost savings for SHI by increasing the efficiency and sustainability of treatment to pure cost stability through a mere resource shift from inpatient to outpatient care and extends to mitigation of further rising future costs. Hospitals would have benefited from the decoupling of money and beds and thus from the freedom gained through integrated care, but would not have to fear any negative financial consequences because of the contractually agreed stable budget.

SHI representatives agreed with hospital representatives on the need for more patient-individual integrated care. They considered the outpatient shift associated with the GTB to be central and desirable. To their opinion outpatient care, actually provided by SHIs accredited physicians and increasingly lacking in several rural areas, was strengthened. SHIs expressed that from an economic point of view the main goal is to stabilise expenditures in order to achieve greater planning security and possible cost savings. Compared to the continuously rising expenses in regular care, expenditure stability was considered as a realistic goal by the firmly negotiated budget, whereas cost savings were estimated as hardly achievable due to the resource shift from inpatient to outpatient sector. Nevertheless, one SHI concluded a contract, whereby any additional costs or savings achieved through integrated care are shared equally between care provider and SHI to create a positive incentive.

#### Compatibility and Complexity

The poor compatibility with processes and structures of regular care and standard remuneration was uttered as relevant by all experts. On the one hand, poor compatibility was explained as caused by the fact that the extensive structural changes in terms of staff, equipment and structural aspects cannot, or only with considerable difficulty, exist in parallel with the regular system. Furthermore, patients from different SHIs would not understand why they receive vastly different care despite same symptoms.

Financial controlling within the model was perceived by the care providers as significantly more complex than by the SHIs. Controllers explained that after DRG introduction in psychiatry in 2012, parallel accounting documentation was agreed in the late adopter regions for control and transparency of individual integrated care services. This enabled the hospitals to have a close-knit overview of the economic effects of integrated care and gave the SHIs the demanded insight into the treatment measures provided.

#### Trialability, Divisibility, Reversibility, and Observability

Trialability and reversibility were consistently rated as extremely poor across all stakeholder groups, although the legal basis should serve as opportunity to try out new ideas. They stated that consistent reorganisation to integrated care in the sense of the GTB would ultimately appear irreversible, as it would be accompanied by multi-layered and profound sustainable changes both in hospitals and stakeholders’ heads that fewer beds suffice.

Especially controllers expressed that at the same time, a GTB has to be inevitably reversible due to the unavoidably contract time limit. They proposed that longer contract terms would thus be desirable for such a comprehensive change in care in order to achieve a higher degree of planning security. They feared that the high investments in, e.g. structural measures, staff training, or fleets of vehicles for home care were associated with considerable economic damage in case of an involuntary return to standard care.

Likewise, psychiatrists commented that the concern about considerable economic damage could prevent a consistent implementation with extensive structural changes, for example in form of a rigorous bed reduction. To counter this risk, some controllers advise a “well-prepared exit strategy”.

Regarding the divisibility, controversial opinions emerge between those contracted with all SHIs and those contracted with a SHI subset. Psychiatrists and controllers expressed to prefer the participation of all SHIs to any insular solutions since the introduction of a parallel system would be more challenging than an overall system conversion. To the opinion of some SHI representatives partial budgets, seen as intermediate stage on the way to a GTB, would have certain advantages due to their fewer actors in the negotiation.

Overall, manifold comments throughout the interviews suggest that observability for interested potential adopters has existed for a long time, established through above-mentioned intensive, informal personal exchange in psychiatrist networks.

#### Resulting Risk

The illustrated lack of sufficient trialability, divisibility and reversibility can be identified as one of the main risks perceived by hospitals.

Representatives of SHIs estimated about SHIs not participating in a GTB that the main risk associated with a GTB would be a loss of control over the psychiatric care actually provided because they had no insight into individual performance with GTB, unlike in previous integrated care contracts that were designed as fee for service.

Representatives of all stakeholder groups rated that this risk could be countered by regular personal exchange and adequate monitoring.

Finally, one actor commented that local conditions, e.g. a possible neighbourhood veto against a GTB, could involve a decisive risk. The involvement of crucial actors in the region and thus interdisciplinary communication would have been promising for contract conclusion.

### Characteristics of Decision-Making Unit

Rogers’ theory sees the course of the diffusion process strongly dependent on the indivuals. Participants from all stakeholder groups rated the GTB-innovators or early adopters, especially on the part of psychiatrists, as well recognised in their circles and as opinion leaders in their networks. When summarising various statements from the material the innovators are also characterised by openness to change and thus great commitment and enthusiasm to realise these changes despite perceived risks. They tend to have a positive attitude towards science, thus enabling accompanying evaluations. Perseverance and acceptance of occasional setbacks can be attributed to them, as their project to convince contractors to implement integrated care enabled by GTB has usually been a long and tough process.

Especially psychiatrists postulated that on the part of the SHIs would be a low degree of willingness to innovate and awareness of necessary change in mental health care. Their restraint towards change and new ideas would lead to the fact that communication messages about the GTB meet with higher resistance, the required venturesomeness would be missing. SHIs commented that they were rather reserved and sceptical about a system change and would prefer a watchful waiting attitude, partly until further evaluation results were available.

Statements from various participants suggest that SHIs’ attitude is largely determined by their board of directors und renewed negotiation attempts after change in leadership seem attractive. However, necessary detailed knowledge of the negotiation or contract history is often tied to individuals so that without a guaranteed transfer of crucial knowledge, a change of personnel or SHIs structures (from local to national organisation or after merger) can become a crucial barrier.

### Communication Behaviour of Decision-Making Unit

Psychiatrists cultivate a close collegial exchange of experience within their networks, also via interpersonal channels, face-to-face exchanges and via scientific publications as already mentioned above. Later adopters uttered that the extensive networking of psychiatrists made it possible that they could easily access the basic principles already developed. In contrast, interviews did not show analogue networking of hospital economists.

A controller stated that communication between hospitals and SHIs were a manifold combination of written exchanges, personal contacts, official negotiation rounds and informal talks. Decisions would strongly depend on the individuals and their personal relationship. Various participants expressed that despite the inevitable particular interests of the actors involved, a certain degree of willingness to compromise were a key for successful contract conclusion. Several uttered that becoming familiar with the contracting partner during the negotiation process has led to greater appreciation and thus to empathy and long-lasting mutual trust. This would result in higher ability to deal together with uncertainty and risks while facing unforeseen challenges. However, experts from the hospitals’ side stated that a high degree of SHI mistrust has led to high, almost unfulfillable demands on the hospitals.

The extent to which the SHIs’ organisational form plays a role in the GTB-diffusion was controversially discussed. The various statements suggest that nationally organised or merged SHI have the advantage of an overview of several similar models with corresponding empirical values from colleagues in other areas. They can also benefit from their opinion leadership compared to smaller SHIs. In contrast, locally anchored SHIs are closer to local contractual partners and conditions. Common to all SHIs was that unclear personnel responsibility was widely perceived as a challenge for further dialogue because of the absence of specialised departments for new integrated care models.

The entire innovation decision-making process is assessed across all stakeholder groups as being significantly and positively influenced by the support of politicians from the federal and state governments. In summary, the political support, which varies greatly from region to region, promotes the likelihood of a positive contract conclusion. Some respondents attribute to state politics the role as a neutral authority mediating in case of differences between negotiating partners. At the same time, politicians pursue their own interests regarding integrated care projects; for example, being basically interested in instruments reducing the beds number in the state hospital plan to limit the general trend of bed growth in Germany.

### Prior conditions of the situation

The development of the zeitgeist in psychiatry was presented by psychiatrists as significant for the willingness implementing an integrated care model. They commented that personal attitudes towards contemporary and adequate psychiatric care would have changed noticeably, especially among hospital psychiatrists: Away from long-term inpatient stays, facing the necessary change towards more participation in social life, among other things through more outpatient and home treatment, strengthening autonomy, voluntariness, and participation.

Most stakeholders rather saw rural locations with a single hospital responsible for obligatory care better suited to implement integrated care with a GTB. Regions with a definable patient catchment area and a stable patients’ number would be advantageous. In principle, a dominant or monopoly position of the local hospital would be favourable. However, urban psychiatrists stated that urban areas could simplify home treatment due to manageable and compressed catchment areas. Others remarked that a competitive situation in local psychiatric care resulting from provider diversity or competitors in the neighbouring care area would complicate implementation, since questions of budget distribution and care responsibility would have to be clarified.

## Discussion

The study identified several factors that explain the stagnant diffusion of the innovative care model. These are mainly: lack of trialability, lack of reversibility, and poor compatibility with existing treatment and remuneration structures. Due to a multi-actor constellation, the process of negotiating and establishing the model is highly complicated. This is coupled with a lack of routine in the development of innovative concepts and accompaniment of their implementation. Diffusion was facilitated in the environment of particularly strong individual actors with a special willingness to take risks, opinion leadership and informal networking. Monopoly position of the hospital, well-defined care region, and non-profit governance were identified as favourable framework conditions.

Innovations’ diffusion in health care is still a major topic in many systems and slowness or failure to diffuse a common outcome [43,44]. According to Rogers’ model, characteristics of the innovation object itself, such as difficult trialability and lack of reversibility, are barriers to rapid diffusion that are not compensated for even by good observability and extensive relative advantages. Innovations that can be split for trial are generally adopted more quickly. While in our study divisibility is rated poorly by most respondents, experience from regions with a parallel system itself shows that this partial implementation with fewer contractors is quite possible and even beneficial. Less complex innovations are also adopted more quickly than those where the adopter must develop new skills and understanding. The pronounced complexity of the GTB may be one reason for the low diffusion rate. It also explains why structural and process innovations in health care – especially disruptive innovations [45] like capitation models – are significantly more difficult than product innovations. Moreover, product innovations are promoted by manufacturer investments, which cannot happen with structural innovations, because there is no bundled business interest behind them.

Generally, the innovation must prove itself in a health policy environment with strong forces of inertia in the regular system. It is characterised not only by institutional complexities and dominating tendencies towards status quo preservation, but even by path dependencies that make disruptive innovations practically impossible [29,46]. Moreover, the incentive in current psychiatric care system lies largely in the expansion of relatively well remunerated inpatient care. Private hospitals, by virtue of their ownership, are inevitabely out to force profit. Municipal providers, however, accept a balanced economic result.

The beneficial characteristics of the actors were found to varying degrees among the participants. While the characteristics of venturesomeness, innovativeness, and willingness to take risks were found on the side of the psychiatrists and the controllers, these were not reported to the same extent among the SHIs. The individuals’ impact is also lower among SHIs with large structural units.

Extensive guidance for the aspects of treatment spectrum as well as descriptions for possible contract designs from the financial point of view are available [11]. But due to the exceedingly low number of SHIs willing to negotiate, it can be assumed that the knowledge of the benefits perceived by the psychiatrists does not sufficiently reach the SHIs – this could be interpreted as a communication problem. Or the benefits were evaluated less convincingly from the SHIs’ point of view because short term economic risks of a GTB dominate over the long-term economic effect of integrated care. However, results of a nationwide evaluation from the SHIs’ perspective might change the SHIs’ strategy. First positive results have been published after completion of our study [17,47].

Diffusion is described by Rogers as a very social process involving interpersonal communication relationships (interconnectedness). Most people do not evaluate an innovation primarily based on scientific studies, but rely mainly on a subjective evaluation of an innovation communicated to them by precursors [48]. This dependence on “near peers” suggests that the heart of the diffusion process is imitation by potential adopters of their network partners in ultimate proximity. This underlines diffusion theory concepts parallel to Rogers [49]. A near peer phenomenon can be seen in one federal state where a large part of the districts implemented a GTB. Von Peter et al reported that compared to models in the rest of the federal republic, these show a more advanced implementation, more pronounced integrated care, more outpatient treatment and stronger support for state politics [50]. The study also confirms our findings of a positive effect of public ownership and lower number of actors. Also, identical persons as stakeholder representatives in the various negotiations might have been involved.

Communication is effective when it takes place within similar actors (psychiatrists with psychiatrists, businessmen with businessmen), but problematic when different circles communicate with each other (psychiatrists to businessmen, hospital to SHIs). The number of actors involved in an innovation decision is negatively correlated with the diffusion rate and the speed of diffusion. This multi-actor constellation is inevitably pronounced in GTB-negotiations. Since successful contracting is only possible if there is complete consensus among vastly unlike groups of actors, contract negotiations are usually complex and in the cases of our study lasted over years. Indeed, current legislation requires contracts to be concluded with SHIs at individual actor level instead of a mandatory association level as happened with early GTBs, which aggravates the multi-actor constellation.

Underadoption has also been described for Assertive Community treatment (ACT), another complex innovation in integrated mental health care with a large number of high-quality effectiveness studies [51]. Although a different theoretical model is used to analyse the diffusion process, the fit of characteristics of the diffusion object into specific settings and to key actors also play an essential role here [51]. Targeted investment in early adopters, making their activity visible and giving them room for change, and acknowledging that adopters of any status need resources for their tasks in the diffusion process are seen as promoting factors [44]. These and other recommendations, however, refer to innovations in circumscribed organisations [52], but may be only transferable to a limited extent to innovations on a macro level.

### Strength and limitations

Main limitation of the study is that sceptical observers i. e. from privately owned hospitals could not be recruited. Therefore, the full spectrum of reasons against the GTB can only be speculated, such as lack of treatment quality with such short inpatient stays, lack of crisis resistance with so few beds, as well as the postulated profit orientation of the GTB-opponents. However, it is plausible that the business model of profit-oriented hospital operators is not compatible with the integrated care model associated with the GTB, although explicitly encouraged by the legal basis.

We used snowballing which helped to obtain a complete set of actors involved per region. The disadvantage is that a circle of basically interested actors could not be left. As we could not compensate for this by additional recruitment efforts a critical perspective on the GTB might remain underrepresented.

Our study did not include basic hospital staff, because from the perspective of Rogers’ model, the persons, who have authority for strategic decisions, play a particularly important role in establishing a new health care model. For various follow-up aspects such as implementation in adopters’ hospitals or patient-related factors the perspective of the staff is undoubtedly highly relevant, but these factors were not part of our research question regarding the preceding decision for implementation.

Within the recruited groups of adopters and failed adopters from rural, urban, and metropolitan areas, saturation of information was achieved so that the final number of interview partners per group does not appear to be a disadvantage. As some of the experts were also active at the policy-making-level, this perspective was captured aditionally.

This qualitative study is the first to apply Rogers’ model to the GTB and thus to find explanatory patterns for lack of diffusion at system level. It proved to be adequate for identifying barriers and facilitating factors for implementing integrated care through a global budget. Ideally, this study complements the recent nationwide quantitative evaluations of economic and patient-related outcomes [17] and studies on the case level [18,19].

## Conclusions

Our investigation of the innovative integrated care model based on a global budget using Rogers’ diffusion of innovation theory suggests that the broad communication of the detailed description of the innovation‘s characteristics needs to be enhanced between the different stakeholder groups as well as partly within the stakeholder groups, all of whom are potential new adopters. This is especially the case for the relative benefits of the innovation as well as for the detailed how-to knowledge. The innovation’s inhibiting complexity might be reduced by dividing it because simpler forms ease trialability and reversibility and attract potential new adopters of an innovation. Furthermore, dissolving the multi-actor constellation by reducing the number of actors initially involved in the decision process could accelerate the diffusion. Finally, the claim of interested adopters to initiate and conclude GTB-negotiations should be strengthened by law so that protracted attempts do not longer fail due to the veto of single actors. For both, support is needed through refined legal frameworks.

The creation of broader scientific evidence, usually convincing SHIs to move, inter alia based on routine data, is indispensable and already on its way.

## Additional File

The additional file for this article can be found as follows:

10.5334/ijic.5940.s1 Supplementary material. Supplementary tables 1 to 3.
