# Supplementary material for: Promoting Integrated Care through a Global Treatment Budget: A Qualitative Study in German Mental Health Care using Rogers’ Diffusion of Innovation Theory
Source: Int J Integr Care. 2021 Nov 30;21(4):27. doi: 10.5334/ijic.5940 (PMC8663745; doi:10.5334/ijic.5940)
Supplement: Supplementary material. — Supplementary tables 1 to 3. [file ijic-21-4-5940-s1.pdf]

Supplementary material to manuscript

Promoting Integrated Care through a Global Treatment Budget - A qualitative study in German mental health care using Rogers' diffusion of innovation theory

Farideh Carolin Afraz, Amyn Vogel, Carsten Dreher, Anne Berghöfer

Suppl. table 1: Self-developed questionnaire based on Rogers' theory on innovation diffusion, exemplary version for psychiatrist – early adopter, late adopter, and failed adopter. Left column contains main categories and subcategories from Rogers' model. Right column contains deducted questions.

**Main and sub-categories**	**Introductory questions**
	A1. Please describe in brief your relation to the Global Treatment Budget (GTB). *[Or outline the interviewee's relation yourself and then have them add to it].*<br>A2. I would like to ask you to highlight your central views on psychiatric care in Germany.
	**Questions related to main research issues**
Stages of innovation decision process:<br>• Knowledge<br>• Persuasion<br>• Decision<br>• Implementation<br>• Confirmation	**C) Adoption/non-adoption**<br>I would now like to come back to the GTB.<br>C1. What did you decide to do in the process so far and why?<br>**D) Adoption process**<br>D1. When did you start developing this care model; when did the first ideas emerge? (Initiator) How did you perceive the idea?<br>D1. When did you first learn about the GTB model? From whom did the initiative come? (Imitator) How did you perceive the idea?<br>D2. What aroused your interest? What motivated you at the beginning?<br>D3. How did you evaluate the model? What information did you use to make your decision to participate/not participate/wait?<br>D4. How did you approach the model yourself? Can you tell us something about the first steps of realisation?<br>D5. When did you install/contract the model? Or rather, when, and why did you decide not to adopt the model?
Prior conditions of the situation:<br>• Social norms	**E) Situational perception**<br>E1. What is expected of *[function of interviewee e.g.]* the head of a psychiatric unit and to what extent does introducing such an innovation fit into this expectation that is placed on you?

• Individual interests<br>• Economic conditions	E2. Where are the operational requirements in the clinics for GTB in the first place? How is the "standard" remuneration perceived?<br><br>E3. Who are the stakeholders involved in the decision process?<br><br>E4. What should the decision-making process be like? Is consensus a sine qua non? What are the consequences of a veto by a partner? Who at least needs to be convinced?
Characteristics of decision-making unit:<br><br>• Coping with uncertainty and risk<br>• Venturesomeness<br>• Socioeconomic status<br>• Cosmopoliteness<br>• Innovativeness<br>• Abstraction capability<br>• Personal values	**F) Characteristics of the actors**<br><br>F1. Did you perceive risks and how did you deal with them?<br><br>F2. What aspects are in the priority for you in a care model?<br><br>F3. How strong do you consider your influence on opportunities for change?<br><br>F4. How difficult did you find it to find supporters for the introduction of a GTB?<br><br>F5. How difficult or easy did you find it to promote the GTB and find appropriate platforms for it?<br><br>F6. How difficult or easy did you find it to transfer examples from other regions to your region?
Communication behaviour of decision-making unit	**G) Communication process**<br><br>G1. How is the communication between the actors in the decision-making process? How quickly is feedback on the success of the innovation given to the GTB user itself and to previous GTB non-adopters and potential regions joining the GTB? Does communication take place through formal or informal channels?<br><br>G2. How extensively did you initially communicate the model and how did you expand the audience?
Perceived characteristics of the innovation:<br><br>• Relative advantage<br>• Compatibility<br>• Complexity<br>• Trialability / divisibility/ reversibility<br>• Observability<br>• Resulting Risk	**H) Characteristics of the diffusion object**<br><br>H1. Can the GTB be run in parallel with regular care in your department?<br><br>H2. How does the billing arithmetic work and what is different about it compared to standard care?<br><br>H3. Have you been able to try out GTB in your department without immediately entering a long-term contractual commitment?<br><br>H4. Is the introduction of GTB reversible?<br><br>H5. How is the communication with the outside world? What do outsiders need to know if they also want to introduce the GTB, and how can this be communicated? What is the communication between the actors in the decision-making process?

	H6. What effort and risk are associated with the introduction?
	**Additional questions**
	B1. Let us imagine that you were allowed to shape psychiatric care in Germany according to your own wishes: Which guiding goals would you define? Which potential guiding goals do you consider problematic or undesirable? (justification)<br>B2. Do you consider the ideas you have just mentioned to be practicable and realisable?<br>B3. Let's come back to your desired guiding objectives: Now this is a context in which you do not decide, act and act alone. With a view to other relevant groups of actors: Which values or perceptions of mental health care do you consider essential, which values and perceptions, e.g. of the other actors do you consider obstructive to the realisation of your guiding goals?
	**Closing the interview**
	J1. With your current level of knowledge and experience, would you make the same decision again?<br>J2. What do you personally think should happen so that more hospitals implement this model?<br>J3. Is there anything else that you think is important that we haven't talked about yet? What else would you like to tell us about the GTB?

Suppl. table 2: Main code categories and subcategories. Main code categories and subcategories marked with ® were operationalised from Rogers' diffusion of innovation model, subcategories marked with * were added inductively from the data generated.

1 ANCHOR EXAMPLES	*133*
**2 STAGES OF INNOVATION DECISION PROCESS®**	
**2.1 Knowledge®**	
2.1.1 Awareness-Knowledge®	10
2.1.2 Principles-Knowledge®/How-to Knowledge®	6
2.1.2.1 Comparable Project/ Personal Experiences*	72
**2.3 Adoption®**	
2.3.1 Re-Invention®	22
**2.4 Decision®**	
2.4.1 Broad Consensus*	27
2.4.2 Adoption® → see Federal political Context*	→67
2.4.3 Waiting and Adoption*	0
2.4.4 Rejection®	15
2.4.4.1 Lack of Leverage*	3
**2.5 Confirmation/ Evaluation of Innovation Consequences®**	38
2.5.1 Permanent Adoption*	0
2.5.2 Adoption and later Discontinuation*	0
**3 CHARACTERISTICS OF DECISION-MAKING UNIT®**	
**3.1 Cope with Uncertainty and Risks®**	7
**3.2 Venturesomeness®**	8
3.2.1 Scepticism/ long Hesitation®	11
3.2.2 Curiosity*	2
3.2.3 Aspiration/ Commitment/ Enthusiasm/ Motivation®	35
3.2.4 Persistence/ Patience/ Acception of occasional Setback®	14
**3.3 Influence®**	23
**3.4 Opinion Leadership®/ Assertiveness®**	50
3.4.1 Cosmopoliteness®/ Networking®	34
**3.5 Interest in new Ideas®/Innovativeness®**	10
**3.6 Abstraction Capability®**	8
**3.7 Fears/ Concerns***	11
**3.8 Professional Skills***	27
**3.9 Favourable Attitude toward Science®**	21
**3.10 Personal Attitude and personal Way of Working***	0
**3.10.1 Advanced Working Method/ Foresight in general ***	5

3.10.1.1 Negotiation-Know How/ Empathy for Negotiating Partners*	7
3.10.2 Traditional Way of Working/ Lack of Foresight in general*	9
**3.11 Rationality***	18
**3.12 Seeking Information about Innovations actively***	6
**4 PERCEIVED CHARACTERISTICS OF THE INNOVATION®**	
**4.1 Relative advantage®**	
4.1.1 Economic conditions: Advantage/ Cost Stability/ Efficiency*	27
4.1.1.1 Positive Incentive*	19
4.1.2 Better Work Situation*	14
4.1.3 Newly gained Freedom/ Flexibility*	36
4.1.3.1 Better, individual Patient Care*	45
4.1.3.1.1 More outpatient instead of inpatient Care*	39
**4.2 Compatibility®**	9
4.2.1 Parallel Structures® → see Divisibility + parallel Structures®	→34
4.2.2 Similar existing Structures and Experiences*	14
4.2.3 Infrastructure (geographical + in terms of psychiatric Care)*	42
**4.3 Complexity®**	18
**4.4 Trialability®**	0
4.4.1 Trialability®	19
4.4.2 Reversibility®	40
4.4.3 Divisibility/ parallel Structures®	34
**4.5 Observability®**	32
4.5.1 Observability in the near and far Environment*	31
4.5.2 → see Confirmation/ Evaluation of Innovation Consequences®	→38
**4.6 Risks®**	73
**4.7 Effort incl. Costs***	57
4.7.1 IT-Controlling*	5
**4.8 Expanding Employee Responsibilities + Establishing a Culture***	49
**4.9 A Means to an End***	4
**4.10 Contract Design/ Negotiation Process/ Expiry/ Duration***	117
4.10.1 Legal Forms® → see Federal Political Context*	→67
4.10.2 Transparency / Transparent Remuneration Structure / Control Mechanisms*	38
**5 COMMUNICATION BEHAVIOUR OF DECISION-MAKING UNIT®**	
**5.1 First Step: Who approached whom and when***	20
**5.2 Second Attempt at Negotiation***	17
**5.3 Groups involved/ Collective Decision of different Actors***	56

5.3.1 Members of federal State* → see Political Context of federal States*	→44
5.3.2 People experienced in Psychiatry*	7
**5.4 Compromise***	12
**5.5 Personal Impression of the Negotiations + personal Interaction***	92
**5.6 Trust/ Mistrust / Appreciation***	42
5.6.1 Control Mechanisms* → see Transparency/ Transparent Remuneration Structure/ Control Mechanisms*	→38
**6 PRIOR CONDITIONS®**	
**6.1 Individual Interests®**	53
**6.2 Economic Conditions/ Costs/ Efficiency®**	19
**6.3 Lack of Interest of Health Insurance Funds***	15
**6.4 Structures and Heterogeneity of Health Insurance Funds***	34
**6.5 Politics***	
6.5.1 Federal Political Context*	67
6.5.2 Political Context of federal States*	44
**6.6 Psychiatric Tourisms***	4
**6.7 Mental Health Care***	
6.7.1 Zeitgeist of psychiatric Care/ social Norms*	21
6.7.1.1 Away from custodial Psychiatry*	11
6.7.2 (Primary) Prevention/ Prophylaxis required*	4
6.7.3 Outpatient Treatment required*	2
6.7.4 Shortage of skilled Workers challenging*	2
6.7.5 Flexibilisation required/ Sector Limits existing*	31
6.7.6 Regular Remuneration of psychiatric Care*	27
6.7.6.1 False Incentives*	41
6.7.7 Suboptimal Care/ Inefficiency*	6
6.7.7.1 Medical Deficiency in rural Areas and office-based Physicians*	7
6.7.7.2 Medical Deficiency (especially of critically ill Patients) + Choice of light and severe Cases*	12
**6.8 Personal Values® towards Mental Health Care**	53
**6.9 Medical Self-Government in Germany***	4

Suppl. table 3: Characteristics of selected mental health care regions included into the study.

**Early adopter**
The first region implemented the GTB to practice integrated care shortly after the pilot region. The rural-structured district has one inpatient service provider under municipal sponsorship. The GTB-contract was concluded with the state association of all SHIs in accordance with the law at that time. The community-based psychiatric care is dedicated to a social psychiatric approach and takes place in basically open treatment areas, both outpatient, day-clinic and inpatient mixed and in home treatment. With detailed knowledge of the care-relevant and contractual design options of a GTB from the pilot neighbourhood, the management of the hospital approached the payers for discussions to join the model itself 2 years later. The GTB was extended in each case under applicable legal conditions. The inpatient area was halved in favour of a strong expansion of integrated day-clinical and outpatient structures.<br><br>The second region nearby is a rural county with urban parts. The only multi-site inpatient psychiatric provider is non-profit and established a GTB as the first successor to the pilot. The contract was with the state association of all SHI. The number of beds was already low under regular care and was hardly reduced under GTB conditions, but day-clinic care was significantly expanded and integrated with home treatment. The model was extended in each case under applicable legal conditions.
**Late adopter**
In the urban region, the hospital is the only psychiatric provider, and non-profit under church sponsorship. The holistic (psychiatric, somatic, social) and community-integrated care principle includes inpatient, day-clinical and outpatient care. The GTB started about 10 years after the early adopters and was concluded with all SHIs. Inpatient treatment days have been reduced somewhat since then.<br><br>In the metropolitan region, mandatory psychiatric care is provided by several hospitals (private, non-profit, and municipal), each for a sub-region. The GTB was agreed between several hospitals of the municipal hospital group and one large German SHI and covers urban hotspot regions. In addition to inpatient, day-clinical and outpatient care, home treatment was integrated as a new service. Inpatient treatment days could be reduced for the insured. The integrated mental health care model could better reach the difficult-to-treat patients.
**Failed adopter**
Both urban regions with a rural setting are served by a non-profit clinic under municipal sponsorship.<br><br>With the knowledge of concrete preceding integrated care models (selectively and temporarily contracted with single SHIs) through active gathering of information, the hospital of the first region, interested in the GTB, worked on a concept to approach the contract partners with it. Over several years, the concepts were presented to the payers and intensively negotiated. Nevertheless, a contract was rejected by the payers for various reasons. No attempt was started to implement the GTB with only a selection of health insurers. During the negotiation period of about 10 years, the number of inpatient beds almost doubled, and the number of day-clinic places quadrupled, while the number of inhabitants increased only discreetly.<br><br>The second hospital offers inpatient, day-care and outpatient psychiatric treatment and is intricately linked with physicians in private practice and providers of psychosocial assistance as well as the public health service in the city. This region has received several awards for its

integrated care model, which also includes work across social insurance codes. GTB-negotiations failed years later after administrative and legal obstacles in the establishment of a new management company to control care centrally were insurmountable.
**Observer**
The large rural region with some urban centres is served by a hospital with an inpatient centre, several day-clinics and community psychiatric sites. It is a public institution owned by the district. For many years, the hospital implemented an integrated care project with a large German SHI. Innovative components included peer support, trialogical work, and home treatment. After several years of negotiations with other SHIs, a GTB between the hospital and almost all statutory and private health insurers was launched in the region in 2020.
